# Near-peer teaching (NPT): the importance of process evaluation

**DOI:** 10.3402/meo.v19.24583

**Published:** 2014-06-10

**Authors:** Cynthia G. Reyes, Rodrigo E. Elizondo-Omaña, Oscar de la Garza, Santos Guzmán

**Affiliations:** Human Anatomy Department, Medicine School, Universidad Autónoma de Nuevo León, Monterrey, México

Dear Editor,

Near-peer teaching (NPT) is an instructional method whereby more advanced students tutor their junior classmates by temporarily assuming the role of instructor. In recent years, this collaborative, student-centered, approach has garnered widespread, international interest – mainly among medical education programs.

Many applications of NPT occur in contexts that use some type of problem-based learning (PBL) and/or small group settings featuring a fairly low teacher-to-student ratio (1). In fact, in such situations, the feasibility of conducting small group discussion sessions without near-peers can be dramatically limited.

Although the feedback from both instructors (tutors) and students involved in NPT, in our experience, tends to be quite positive, careful evaluation and monitoring of the process is crucial to promptly identify any issues that may interfere with achieving the stated learning objective(s). Furthermore, the development of such mechanisms requires detailed documentation and evaluation to ensure that resulting changes have the desired (positive) effects.

In our department, we have used NPT for more than five years and have identified several problems in the implementation process; for example, during the discussion sessions of clinical cases (1), the interaction between the instructor and students can be distorted – diverting attention away from the targeted learning objective. Therefore, we developed a monitoring system of training sessions, monitor tutors, satisfaction surveys, and specific rubrics to evaluate the NPT implementation process.

Associated training sessions for instructors (tutors) occurred weekly, and stressed three main objectives: 1) understanding the learning objective of the session, 2) reviewing the adequacy, relevance, and approach of the thematic content, and 3) understanding the function of the instruction process. The monitor tutors were present in all discussion activities and charged with detecting and correcting any situation or anomaly *as it occurred* within the session.

The subsequent satisfaction surveys then elicited the opinions of all NPT participants (professors, instructors, and students) to validate the impact of the changes on program function. Finally, we use specifically developed rubrics to evaluate the participation of students in the discussion activities. In summary, although there is more to be done, a keen eye must always be kept on process to ensure that positive strides are made to continually maximize the effects of this approach.
